# The expression of *Drosophila melanogaster Hox* gene *Ultrabithorax* is not overtly regulated by the intronic long noncoding RNA *lncRNA:PS4* in a wild-type genetic background

**DOI:** 10.1093/g3journal/jkab374

**Published:** 2021-11-15

**Authors:** Anita Hermann, Dave Kosman, William McGinnis, Ella Tour

**Affiliations:** Section of Cell and Developmental Biology, Division of Biological Sciences, University of California, San Diego, La Jolla, CA 92093-0355, USA

**Keywords:** long noncoding RNA, lncRNA, *Hox* gene, *Ultrabithorax*, *Ubx*, *Drosophila melanogaster*, gene regulation, *Contrabithorax* (*Ubx^Cbx-1^*) homeotic allele

## Abstract

Long noncoding RNAs (lncRNAs) have been implicated in a variety of processes in development, differentiation, and disease. In *Drosophila melanogaster*, the bithorax *Hox* cluster contains three *Hox* genes [*Ultrabithorax* (*Ubx*), *abdominal-A*, and *Abdominal-B*], along with a number of lncRNAs, most with unknown functions. Here, we investigated the function of a lncRNA, *lncRNA:PS4* that originates in the second intron of *Ubx* and is transcribed in the antisense orientation to *Ubx*. The expression pattern of *lncRNA:PS4* is complementary to *Ubx* in the thoracic primordia, and the *lncRNA:PS4* coding region overlaps the location of the large insertion that causes the dominant homeotic mutation *Contrabithorax-1* (*Ubx^Cbx-1^*), which partially transforms *Drosophila* wings into halteres via ectopic activation of *Ubx*. This led us to investigate the potential role of this lncRNA in regulation of *Ubx* expression. The *Ubx^Cbx-1^* mutation dramatically changes the pattern of *lncRNA:PS4*, eliminating the expression of most *lncRNA:PS4* sequences from parasegment 4 (where Ubx protein is normally absent) and ectopically activating *lncRNA:PS4* at high levels in the abdomen (where *Ubx* is normally expressed). These changes, however, did not lead to changes in the *Ubx* embryonic transcription pattern. Targeted deletion of the two promoters of *lncRNA:PS4* did not result in the change of *Ubx* expression in the embryos. In the genetic background of a *Ubx^Cbx-1^* mutation, the *lncRNA:PS4* mutation does slightly enhance the ectopic activation of Ubx protein expression in wing discs and also slightly enhances the wing phenotype seen in *Ubx^Cbx-1^* heterozygotes.

## Introduction

Long noncoding RNAs (lncRNAs) are a ubiquitous group of RNA transcripts that are over 200 nucleotides long, do not code for potential proteins or peptides longer than 100 amino acids, and are transcribed from a variety of genomic locations, including enhancers, promoters, and intergenic regions (Rinn and Chang 2012; Yamamoto and Saitoh 2019). Such lncRNAs have diverse roles in development, differentiation, and disease (Yao *et al.* 2019). lncRNAs are primarily localized in the nucleus where they can regulate gene expression by a variety of mechanisms, including interactions with enzymatic complexes that modify and remodel chromatin, direct transcriptional regulation via interaction with Preinitiation Complex, and regulation of alternative splicing (Squillaro *et al.* 2020). One of the classic examples of regulatory lncRNAs is *Xist* which controls female X chromosome inactivation in mammals (Yao *et al.* 2019). Another example is the partial derepression of human and mouse *HoxD* genes by mutations in the lncRNA *HOTAIR* (Rinn and Chang 2012; Li *et al.* 2013), although other studies that ablated *Hotair* function in mice reported little or no changes in *Hox* gene embryonic patterning function (Schorderet and Duboule 2011; Lai *et al.* 2015; Amândio *et al.* 2016; Yao *et al.* 2019).

Numerous lncRNAs have been detected within the clusters of *Hox* genes—evolutionarily conserved complexes of genes coding for homeobox-containing transcriptional factors that control axial patterning in bilateral animals (McGinnis and Krumlauf 1992; Kumar and Krumlauf 2016). These lncRNAs are transcribed from both coding and noncoding strands of the *Hox* gene clusters and can be found within *Hox* introns, in intergenic regions, or flanking the *Hox* gene clusters (Kumar and Krumlauf 2016). They can function both in *cis*, by regulating the adjacent *Hox* genes and in *trans*, by regulating the expression of *Hox* genes in other *Hox* clusters or by regulating or modulating the activity of non-*Hox* genes (Kumar and Krumlauf 2016).

The *Drosophila* bithorax *Hox* cluster (BX-C) contains three *Hox* genes: *Ultrabithorax* (*Ubx*), *abdominal-A*, and *Abdominal-B*, as well as many noncoding RNAs (Lipshitz *et al.* 1987; Sanchez-Herrero and Akam 1989; Cumberledge *et al*., 1990; Bae *et al*., 2002; Pease *et al.* 2013; Schor *et al.* 2018). Some of these noncoding RNAs are processed into microRNAs (miRNAs) that can regulate *Hox* expression (reviewed in Garaulet and Lai 2015), but most have no known function. An example of such a non-miRNA-containing lncRNA is *bxd*, which is encoded in upstream regulatory regions of the *Ubx* gene. Functionally, the *bxd* RNA has been reported to repress early transcription of *Ubx* in cis, perhaps by readthrough through the *Ubx* promoter (Petruk *et al.* 2006); however, the ablation of *bxd* RNA revealed that the early transcription repression of *Ubx* transcription in the *bxd* expressing region was very transient and no embryonic or adult *Ubx* mutant phenotypes were detected from *bxd* RNA elimination (Pease *et al.* 2013).

In 2013, Pease *et al.* (2013) discovered novel lncRNAs in a 143-kb region of the *Ubx* locus that included the *Ubx* transcription unit and its upstream *bxd cis*-regulatory region. One of these new lncRNAs originates in the second intron of *Ubx* and is transcribed in the antisense direction relative to *Ubx* (Pease *et al.* 2013), placing it among the Natural Antisense Transcript (NAT) group of noncoding RNAs (Faghihi and Wahlestedt 2009). The early embryonic domain of expression of this lncRNA was in parasegment 4; therefore, Pease and colleagues named it *PS4*. In accord with standard *Drosophila* gene nomenclature (Flybase 2020), we call this transcript *lncRNA:PS4*. Previous studies have shown that in numerous instances the NAT members of the sense–antisense gene pairs regulate the genes expressed from the sense strand (Faghihi and Wahlestedt 2009), raising the question whether this antisense transcript has a role in *Ubx* regulation. Intriguingly, the *lncRNA:PS4* promoter mapped in the vicinity of the *Contrabithorax^1^* (*Ubx^Cbx-1^*) insertional mutation (Lewis 1955; Bender *et al.* 1983). *Contrabithorax* is a dominant mutation that results in a transformation of some adult structures of the second thoracic segment (T2) into those of the third thoracic segment (T3), *e.g.*, transforming wings into halteres (Lewis 1963). The *Ubx^Cbx-1^* allele results from an insertion of 17 kb from a *Ubx* upstream regulatory element into the second intron of *Ubx* and is the only known *Contrabithorax* allele that maps to that region (Bender *et al.* 1983; Lewis 1955). This inverted insertion contains parts of two *Ubx* upstream regulatory regions: 16 kb of the *pbx* region and 1 kb of *bxd*. In *Contrabithorax* mutants, *Ubx* transcript and protein expression are abnormally activated in the wing discs, primarily in the posterior compartment of the disc, which results in a partial transformation of wing toward haltere morphology (White and Akam 1985); however, the mechanisms by which *Cbx* mutations ectopically activate *Ubx* expression are not understood.

Taken together, the *lncRNA:PS4* domain of early expression that is adjacent to the *Ubx* expression domain and the proximity of the *Ubx*-deregulating mutation *Ubx^Cbx-1^* to *lncRNA:PS4* gene lead us to investigate the following questions:

- Does lncRNA:PS4 have a role in the regulation of Ubx transcription?
- To what extent does the Ubx^Cbx-1^ mutation change lncRNA:PS4 transcription?
- If such changes occur, do they play a role in modifying the morphological functions provided by Ubx?

## Materials and methods

### Determination of the extent of *lncRNA: PS4* transcription unit

We used 5′ and 3′ RACE [5′/3′ RACE kit, second generation (Roche)] to determine the start and the end of *lncRNA:PS4* transcription. The extent of the *lncRNA:PS4* transcription unit was determined as follows: the region between the *lncRNA:PS4* promoter and *Ubx* transcription start site was virtually subdivided into regions of about 1 kb long. These regions were cloned by PCR amplification from the genomic DNA of *w^1118^* flies. Antisense RNA probes were generated using *in vitro* transcription and the presence of transcripts from each region was assessed using whole-mount *in situ* hybridization. To determine if any of the transcribed regions were spliced, RT-PCR of 900–1100 bp amplicons spanning the entire predicted transcript was produced, followed by sequencing. For RT-PCR template generation, total RNA was extracted with RNAeasy mini kit (Qiagen) and treated with Turbo DNA-free kit (Ambion) to remove DNA.

### Determination of *lncRNA: PS4* expression pattern

**Figure 1:**
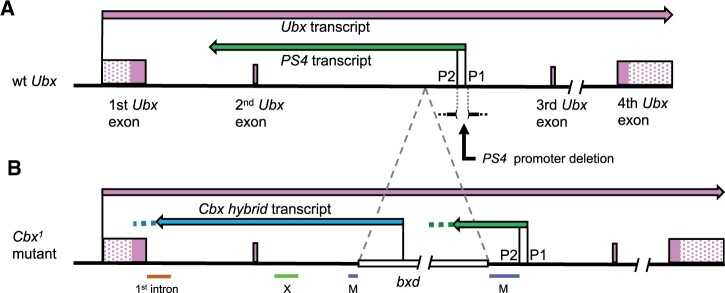
Schematic representation of *lncRNA:PS4* transcription unit within the *Ubx* locus in wild type (A) and *Ubx^Cbx-1^* mutant (B) embryos. *Ubx* exons are indicated as purple boxes; the second and the third exons are alternatively spliced; shading in the first and the last exons indicate untranslated regions. (A) In wild-type embryos *lncRNA:PS4* transcripts (green) originate from the second intron of the *Ubx* gene, driven by two alternative promoters (P1 and P2). *lncRNA:PS4* is transcribed in antisense direction relative to *Ubx* transcripts, contains no introns, and is terminated in the first intron of *Ubx*. The *lncRNA:PS4* promoter deletion eliminates both P1 and P2 promoters. (B) In *Ubx^Cbx-1^* mutants, a 17 kb insertion that contains *pbx* and *bxd* regulatory elements maps ∼1.1 kb downstream of *lncRNA:PS4* transcription start sites, and produces an additional, new hybrid transcript (blue)*.* Note that the *Ubx^Cbx-1^* hybrid transcript terminates closer to the transcription start of *Ubx* than the wild-type *lncRNA:PS4* transcript. Locations of sequences in the *in situ* hybridization probes *M, X*, and *first intron* are indicated.

Whole-mount *in situ* hybridization was performed as previously described (Kosman *et al.* 2004). The *Ubx* cDNA probe was generated from the BSUbx1a plasmid (Harding *et al.* 1985). Another probe to detect *Ubx* nascent transcripts was prepared with a pBluescript plasmid clone that included the 5′-most 1.5 kb of the first intron of *Ubx*. The *wingless* probe was generated from a 3 kb clone in pBluescript plasmid (a gift from B. Cohen). *lncRNA:PS4* transcription was detected via the *M* probe (upstream of *Ubx^Cbx-1^* insertion), or the *X* probe (downstream of *Ubx^Cbx-1^* insertion, Figure 1B). Images were obtained using Leica SP4, Leica SP5, and Leica SP8 fluorescent confocal microscopes and processed in Adobe Photoshop software.

### CRISPR/Cas9-generated mutations in *lncRNA:PS4* promoters

To generate a targeted deletion of the sequences encompassing both putative promoters of *lncRNA:PS4*, two gRNA sequences were designed using CRISPR Optimal Target Finder (Gratz *et al.* 2014) and cloned into *pCFD4-U6:1_U6:3tandem-gRNAs* (Addgene plasmid No. 49411), using the protocol described in Port *et al.* (2014). The first guide RNA sequence targeting the site 182 bp upstream of the “upstream” promoter was placed under the control of the U6:1 promoter in *pCFD4* vector and its sequence was GGAGTAAATTTATCTGGCTCT (in the genome, this sequence would be followed by a 3′ PAM: CGG). The guide RNA sequence targeting the “downstream” promoter was placed under the control of the U6:3 promoter and its sequence was GGTTCATTTCATTTGCCCAA (in the genome, this sequence would be followed by a 3′ PAM: CGG). gRNA cloning was verified by sequencing.

Transgenic lines carrying the gRNA construct were generated by BestGene Inc (Chino Hills, CA, USA). Males of this gRNA line [genotype (y1sc*v1; P{CaryP-v+}attP40/CyO] were crossed to females carrying *Vasa-Cas9* transgene either on the X chromosome (Bloomington stock No. 52669) or on the third chromosome (Bloomington stock No. 51324) and the progeny of these crosses was back-crossed. To screen for the presence of the desired deletions, we PCR-amplified the region surrounding the *lncRNA:PS4* promoters from whole-fly genomic DNA, followed by sequencing of the PCR fragments. One line of transgenic flies was isolated from the cross to a female with a *Vasa-Cas9* transgene on the third chromosome. This line has a 324 bp deletion that eliminates both the upstream promoter and the downstream promoter of *lncRNA:PS4*. Homozygotes for this deletion chromosome survive to adulthood at 5% the expected frequency, although this effect on viability does not map to the 324 bp deletion or to the *Ubx* region, as the chromosome with the small deletion is fully viable when crossed to a chromosome bearing the *Ubx^109^* mutation [*Df(3R)Ubx^109^*^*]*^, which deletes the entire *Ubx* locus.

### *Drosophila* stocks and crosses

The adult wing phenotype of *lncRNA:PS4* heterozygote mutants was assessed in *lncRNA:PS4ΔP/TM3Sb* flies, maintained as a stock*.* Ubx protein expression in the wing discs was assessed in *lncRNA:PS4ΔP/TM3Sb, Kr-GFP* flies, maintained as a stock. *Ubx^Cbx-1^* mutant flies [*Ubx^Cbx-1^/T(2; 3)ap^Xa^, ap^Xa^*] were obtained from Bloomington Drosophila Stock Center (stock No. 3433). This fly strain has had the deletion for the *pbx/bxd* region crossed away from the insertional *Ubx^Cbx-1^* mutation, so is wild type for sequences upstream of the *Ubx* transcription unit. *Ubx^Cbx-1^* adult wing phenotypes were assessed in *Ubx^Cbx-1^/TM3Sb* flies that were obtained by crossing *Ubx^Cbx-1^/T(2; 3)ap^Xa^, ap^Xa^* flies to *lncRNA:PS4ΔP/TM3Sb* flies; the resulting *Ubx^Cbx-1^/TM3Sb* flies were maintained as a stock. Ubx protein expression in the wing discs of *Ubx^Cbx-1^* heterozygotes was assessed in *Ubx^Cbx-1/^TM3Sb, Kr-GFP*, obtained via the cross of *Ubx^Cbx-1^/T(2; 3)ap^Xa^, ap^Xa^* flies to the *lncRNA:PS4/TM3Sb Kr-GFP* flies*. Ubx^Cbx-1^/TM3Sb* and *Ubx^Cbx-1^/TM3Sb, Kr-GFP* flies had identical wing phenotypes*. Pc^3^* mutant flies [*ln(3R)P(Pc^3^),Pc^3^/TM1*] were obtained from Bloomington Drosophila Stock Center (stock No. 106475). *Ubx^Cbx-1^/Pc^3^* flies were obtained via a cross between *ln(3R)P(Pc^3^),Pc^3^/TM1* and *Ubx^Cbx-1^/ap^Xa^, ap^Xa^;* the resulting *Ubx^Cbx-1^/Pc^3^* flies were maintained as a stock.

## Results

### Characterization of *lncRNA:PS4* transcription unit

*lncRNA:PS4* was identified by Pease and colleagues in a systematic survey of noncoding RNAs originating from the *Ubx* gene and its upstream regulatory elements (Pease *et al..* 2013). Pease and colleagues characterized the *lncRNA:PS4* transcription unit as spanning ∼15 kb and including nonprotein coding sequences from the first and the second *Ubx* introns (Pease *et al.* 2013). Here, we characterized the exact boundaries of the *lncRNA:PS4* transcription unit, using 5′ and 3′ RACE, followed by verification using fluorescent *in situ* hybridization with ∼1 kb probes that mapped throughout the *lncRNA:PS4* region (Figure 1).

Using 5′ RACE, we determined that *lncRNA:PS4* has two transcription start sites (P1 and P2), separated by 116 bp and located 14,299 and 14,182 bp downstream of the transcription start site of *Ubx*, respectively. Promoter site 2 is preceded by a good match, TCATTT, to the *Drosophila* InR promoter consensus sequence of TCAGTY (Ngoc *et al.* 2019, Supplementary Figure S1). Promoter 2 (P2, Figure 1) also includes a putative DPE promoter sequence (Supplementary Figure S1). Promoter 1 (P1) contains a weaker match, TCACTG, to the InR promoter consensus and no matches to a DPE consensus sequence (Supplementary Figure S1). In our 5′ RACE reactions, the genomic coordinate for the first 5′ nucleotide of the P1 transcripts is 16,720,127, and for P2 the initiation site maps to nucleotide 16,720,245 (*Drosophila melanogaster* genome release r6.37; Larkin *et al.* 2021). The 3′ RACE results indicated that *lncRNA:PS4* transcripts are polyadenylated and located the 3′ end of *lncRNA:PS4* transcripts to nucleotide 16,729,960 on Chromosome 3R (*D.* *melanogaster* genome release r6.37; Larkin *et al.* 2021). The predicted length of *lncRNA:PS4* transcription unit was 9833 bp for transcripts starting from promoter P1 and 9715 bp for transcripts starting from promoter P2.

To determine if any of the sections of the predicted transcript were spliced, we performed RT-PCR of 900–1100 bp amplicons spanning the entire predicted transcript, followed by sequencing. All RNA-generated sequences were the same size and sequence as genomic DNA, indicating that the *lncRNA:PS4* transcript contains no introns. The only potential protein coding region identified by PhyloCSF in the *lncRNA:PS4* transcript could in theory encode a 39 amino acid peptide, MLQMPTVKTKVPCCHFMNCFCSVGKSTTLTLATHKVLPS (Lin *et al.* 2011). We were unable to detect the conservation of this protein coding region in other *Drosophila* species. No palindromic miRNA precursor sequences were identified in the *lncRNA:PS4* transcription unit.

### *lncRNA:PS4* expression

**Figure 2:**
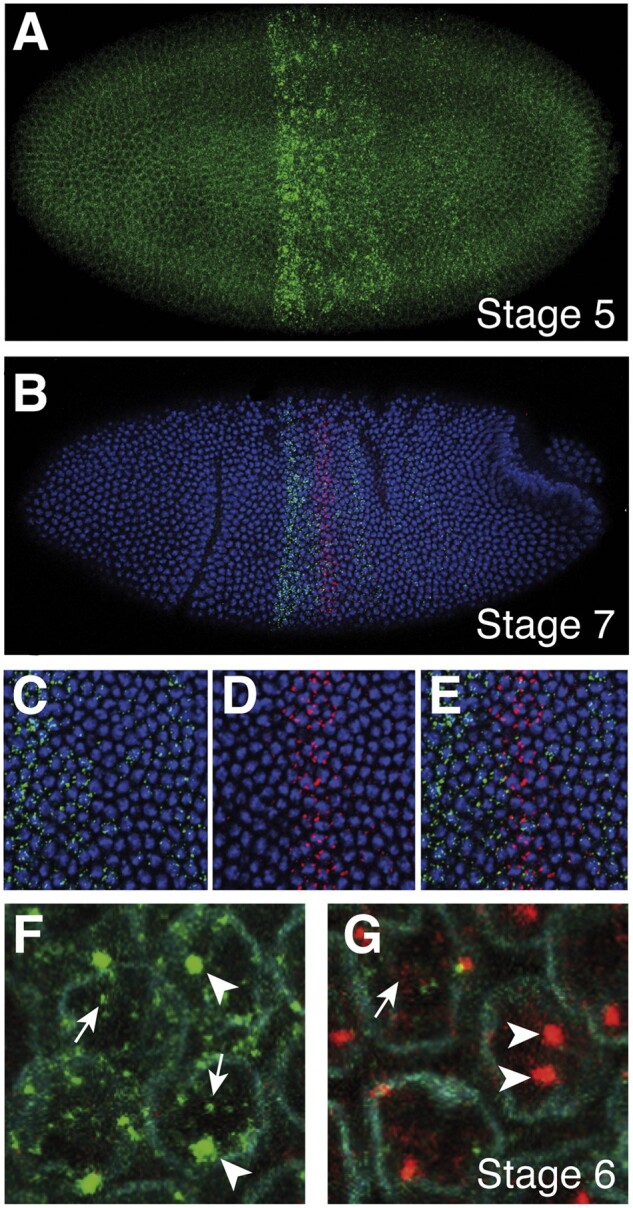
Embryonic pattern and nuclear localization of *lncRNA:PS4* transcripts. (A) *lncRNA:PS4* transcription is first detected at stage 5 by *in situ* hybridization with probe. (B) A stage 7 embryo was hybridized with *lncRNA:PS4* (probe *X*, green) and *Ubx* (*first intron* antisense probe, red) probes and imaged at 20× magnification. In (C–E), the embryo in (B) was imaged at 63× magnification, focusing on the anterior stripes of *lncRNA:PS4* and *Ubx;* maximal projections spanning most of the depth of the epidermis are shown. (C) *lncRNA:PS4* transcripts, (D) *Ubx* transcripts, and (E) an overlay of *lncRNA:PS4* and *Ubx* signals. (F, G) Stage 6 wild-type embryo, probed with *lncRNA:PS4* (probe *X*, green) and *Ubx* (*first intron* antisense probe, red) probes, with the addition of a lamin antibody stain (cyan) that outlines nuclei. (F) Nuclei in the anterior stripe of *lncRNA:PS4* expression, in a region where *Ubx* expression is absent*.* (G) Same embryo as in F, nuclei where *lncRNA:PS4* and *Ubx* expressions overlap. Arrowheads point to nascent transcripts, whereas arrows point to nuclear “specks,” which we attribute to nuclear transcripts (in the case of *lncRNA:PS4)* that are not associated with the transcription site on the *lncRNA:PS4* locus or to transient products of the spliced first intron (in the case of *Ubx*).

Pease *et al.* (2013) described the blastoderm expression of *lncRNA:PS4* as a broad stripe, with PS4 and PS10 as its anterior and posterior borders, respectively*.* Here, we show that *lncRNA:PS4* RNA is first detected by *in situ* hybridization during stage 5 of *Drosophila* embryonic development (Figure 2A). The expression pattern of *lncRNA:PS4* between stages 6 and 12 was previously characterized (Pease *et al.* 2013). During stages 6 and 7, the *lncRNA:PS4* pattern of expression resolves into a broad and strong anterior domain and weaker posterior stripes (Figure 2B and Supplementary Figure S2). Costaining with a *wingless* probe at stage 6 allowed us to map the broad anterior domain of expression to the posterior part of parasegment 4 and the entirety of parasegment 5 (segment T2 and the anterior compartment of T3; Supplementary Figure S3), thus abutting the anterior boundary of *Ubx* expression. *lncRNA:PS4* is excluded from the ventral mesoderm primordia at this stage (Pease *et al.* 2013).

We were also interested in precisely mapping the *lncRNA:PS4* transcription domain relative to that of *Ubx*, in whose intron *lncRNA:PS4* resides. Throughout stages 6–10, *Ubx* and *lncRNA:PS4* transcription domains were largely nonoverlapping; however, in a few nuclei, both *lncRNA:PS4* and *Ubx* transcription were detected in parasegments 5 through 12 (Supplementary Figures S2 and S3). High-resolution imaging at stage 7 (Figure 2, B–E) shows that in the domain of strong *Ubx* transcription, *lncRNA:PS4* transcripts are absent from almost all *Ubx* transcribing nuclei (Figure 2, C–E).

To better understand the cellular localization of *lncRNA:PS4* transcripts, we performed high-resolution imaging of stage 6 embryos after *in situ* hybridization with *lncRNA:PS4* and *Ubx* probes and costaining with lamin antibodies that outline the nuclear envelope (Figure 2, F and G). *lncRNA:PS4* signals are localized almost exclusively in the nuclear compartment (Figure 2F). There is a prominent signal corresponding to *lncRNA:PS4* nascent transcripts (Figure 2F, arrowheads), as well as other small *lncRNA:PS4* nuclear RNA signal “specks” that are not associated with the *lncRNA:PS4* locus, presumably originating from polyadenylated transcripts that accumulate in that compartment (Figure 2B, arrows). *Ubx*, which was detected with a probe against its first intron, was mostly observed as the nascent transcripts being made from *Ubx* loci on the two homologous chromosomes (Figure 2G, arrowheads).

Using *in situ* hybridization, we made multiple attempts to detect spatially localized transcription from *lncRNA:PS4* in wing, haltere and leg discs, using our most sensitive method of tyramide amplification. We never observed a localized signal within discs, or between different discs. The positive controls were *in situ* hybridizations with *engrailed* and *Ubx* cDNA probes, which had easily detectable localized signals. When using quantitative RT-PCR with gene-specific primers covering region *X* (Figure 1B), we did detect low-level transcripts from this region in the discs we tested (wing, leg, and haltere; Supplementary Table S1). The abundance of these low-level transcripts was slightly higher in haltere and leg discs than in wing discs, the opposite of what we expected from the embryonic expression pattern of *lncRNA:PS4*. The positive control was *Ubx* expression levels using quantitative RT-PCR on RNA from the same discs.

### *lncRNA:PS4* location relative to *Ubx^Cbx-1^* insertion

In order to determine the exact location of *Ubx^Cbx-1^* insertion in the *lncRNA:PS4* region, we performed genomic PCR amplification followed by sequencing analysis of the approximate region of *Ubx^Cbx-1^* insertion in *Ubx^Cbx-1^*/T(2; 3)ap^Xa^, ap^Xa^ flies (Bloomington). This allowed us to map the *Ubx^Cbx-1^* insertion location to position 16,721,339 of Chromosome 3R (*D.* *melanogaster* genome release r6.37; Larkin *et al.* 2021). Thus, the *Ubx^Cbx-1^* insertion is located within the *lncRNA:PS4* transcribed region, 1.06 kb downstream of the *lncRNA:PS4* P2 transcription start site.

### The effect of the *Ubx^Cbx-1^* insertion on transcription of the *lncRNA:S4* region

**Figure 3:**
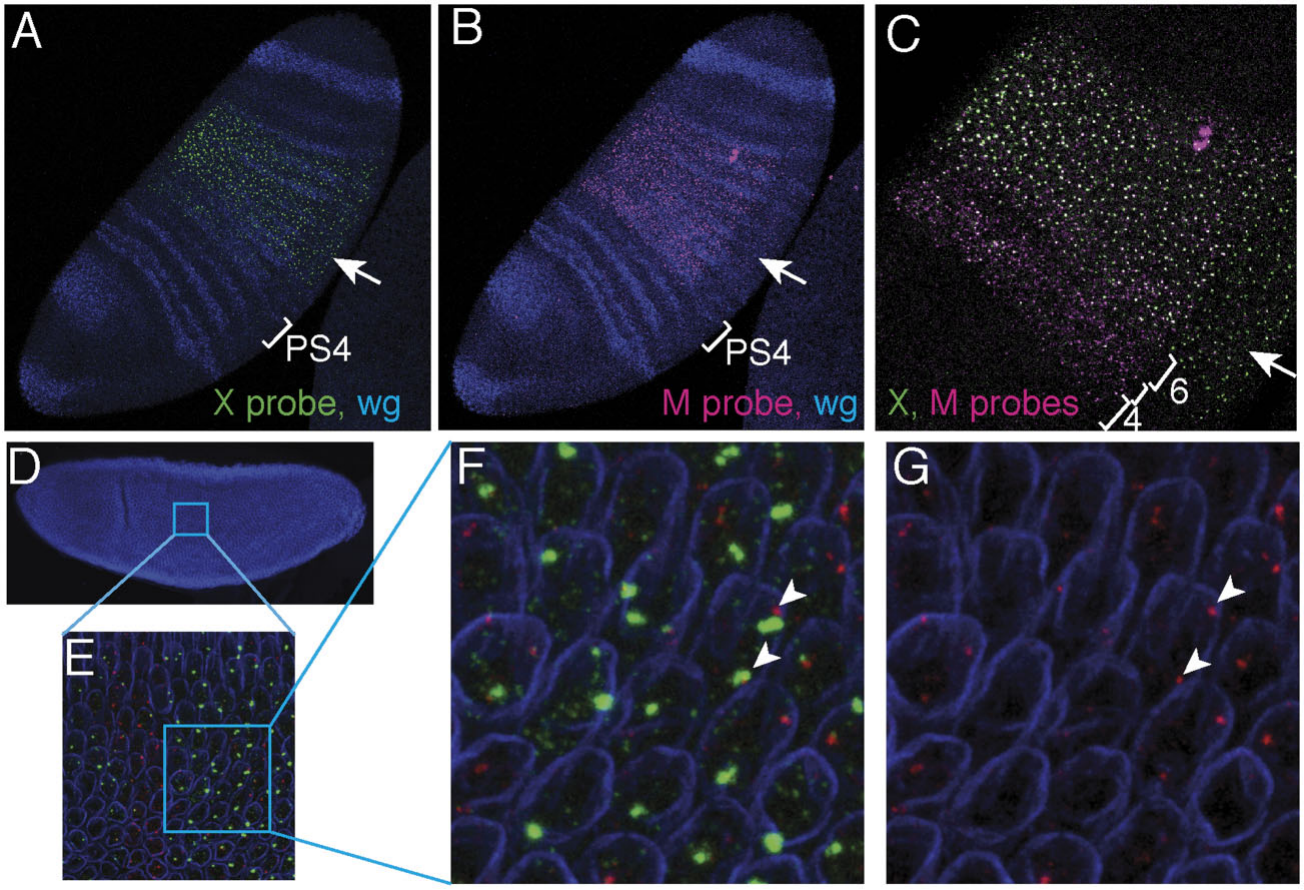
The *Ubx^Cbx-1^* insertion changes the transcription pattern of *lncRNA:PS4* and generates an additional hybrid transcript containing 3′ sequences of *lncRNA:PS4* driven by the *bxd* promoter. (A–C) Stage 6 homozygous *Ubx^Cbx-1^* embryo hybridized with *lncRNA:PS4* probes *X* (green) and *M* (magenta), and a *wingless* probe (blue). (A) The *Cbx hybrid* transcript is detected with probe *X* primarily between parasegments 6–12, and includes the mesoderm primordia (arrow). PS4 denotes fly parasegment 4. (B) The upstream *M* probe detects *lncRNA:PS4* transcripts in parasegments 4 and 5 and in the broad pattern overlapping with the *Cbx hybrid* transcript and is largely excluded from the mesoderm (arrow). (C) Overlay of *X* and *M* signals. Parasegments 4, 5, and 6 are indicated with brackets, parasegments 4 and 6 are labeled 4 and 6, respectively. Mesoderm is indicated with an arrow. (D–G) Early stage 6 *Ubx^Cbx-1^* mutant embryo, detecting early *Ubx* transcription (*first intron* probe, red), along with the *Cbx hybrid* transcript (*X* probe, green), and lamin stain (blue). (D) 10× magnification, lamin stained embryo. (E) 63×magnification of the area marked as a square in (D). (F, G) Magnified image of the area marked as a square in (E). (F) Overlay of *Ubx* and the *Cbx hybrid* transcripts (G) *Ubx* transcripts only. Arrowheads point to *Ubx* and *Cbx hybrid* transcripts located at the same site. Location of the *M, X*, and *first intron* probes is shown in Figure 1B. *M* probe corresponds to *lncRNA:PS4* sequences 1.4 kb upstream of the *Ubx^Cbx-1^* insertion and 0.4 kb downstream of it.

The *Ubx^Cbx-1^* insertion profoundly changes the extent and the pattern of *lncRNA:PS4* embryonic transcription (Figure 3, A–C). The insertion splits the *lncRNA:PS4* transcription unit and results in two separate transcripts, as shown schematically in Figure 1B: a transcript containing ∼1 kb of 5′ *lncRNA:PS4* sequences fused to an unknown amount of *Cbx^1^* insertion sequence, and another transcript initiated within *Ubx^Cbx-1^* insertion that is fused to the 3′ most 8.6 kb of *lncRNA:PS4*. The homozygous *Ubx^Cbx-1^* mutant embryo shown in Figure 3, A–C demonstrates the different expression patterns of these two transcripts. In this *in situ* hybridization, *lncRNA:PS4* transcription was imaged with two probes (Figure 1B): probe *M* contains 1.4 kb of sequence upstream and 0.4 kb downstream of the *Ubx^Cbx-1^* insertion (Figure 3B) and probe *X*, located downstream of the insertion, thus detecting the transcription of the transcript that initiates within the *Ubx^Cbx-1^* insertion and has 3′ sequences of *lncRNA:PS4* (Figure 3A). To simplify, we will refer to this transcript as the *Cbx hybrid* transcript.

The strongest domain of the *Cbx hybrid* expression was posterior to parasegment 6 and its expression extended uniformly to the posterior boundary of parasegment 12 (Figure 3A). This transcript was also observed in a few nuclei located in parasegments 4–6 (Figure 3, A and C). In addition to the abundant posterior expression relative to wild-type *lncRNA:PS4*, the *Cbx hybrid* transcript was not excluded from embryonic mesoderm, unlike wild-type *lncRNA:PS4* (Figure 3, A and C, arrow). The *Cbx hybrid* transcript pattern mimics the normal expression pattern of the *bxd* transcript (Lipshitz *et al.* 1987; Pease *et al.* 2013; Petruk *et al.* 2006). In *Ubx^Cbx-1^* mutants, the *bxd* promoter and regulatory sequences lie within the 17 kb *Ubx^Cbx-1^* insertional mutation, in the opposite orientation to their normal direction in the BX-C complex. The expression of the *Cbx hybrid* transcript, therefore, appears to be driven by *bxd* enhancers located in the insertion. In the hybrid transcript, transcription of the 3′ region of *lncRNA:PS4* extends beyond its normal termination site to at least the vicinity of the *Ubx* first exon, since *Cbx hybrid* transcripts can be detected with a probe corresponding to the 5′ most 1.5 kb of the first intron of *Ubx* (Supplementary Figure S4, A and B).

The *M* probe primarily detected the expression of the *lncRNA:PS4* that is encoded upstream of the *Cbx^1^* insertion (Figure 3B). This 5′ 1 kb region of *lncRNA:PS4* continues to be transcribed in parasegments 4 and 5 and continues to be largely excluded from the mesoderm, similar to the *lncRNA:PS4* in wild-type embryos (Figure 3B, arrow). Because *M* probe has a small amount of homology to sequences downstream of the *Cbx^1^* insertion (Figure 1B), it also weakly detects the *Cbx hybrid* transcript (see the weak mesodermal signal, probably resulting from codetection of the *Cbx hybrid* transcript in Figure 3, B and C). Thus, in *Ubx^Cbx-1^* mutants, the *lncRNA:PS4* transcription unit is split into two transcripts: the 1 kb upstream of the insertion that is expressed in the largely wild-type *lncRNA:PS4* pattern and another, containing over 8 kb of *lncRNA:PS4* downstream of the insertion that is expressed in *bxd* pattern (Figure 3C).

As the *Ubx^Cbx-1^* insertion eliminates the parasegment 4 and 5 expression of the 3′-most 8.6 kb of *lncRNA:PS4* transcribed region, we next investigated whether this change was associated with the expansion of *Ubx* transcription into that region. We detected no expansion of embryonic *Ubx* transcription into parasegments 4 and 5 of *Ubx^Cbx-1^* mutants (Supplementary Figure S4, C and D), suggesting that PS4–PS5 expression of *lncRNA:PS4* was not necessary for the repression of *Ubx* transcription anterior to its normal domain in embryos. Furthermore, the abundant and broadened transcription of the 3′ portion of *lncRNA:PS4* sequences in parasegments 6–12 of *Ubx^Cbx-1^* mutants were not associated with suppression of *Ubx* transcription in those parasegments (Supplementary Figure S4, C and D).

Using high-resolution microscopy, we investigated whether the presence of the *Cbx hybrid* transcript interferes with transcription of the *Ubx* gene from the same locus. To that end, we detected *Ubx* transcription using a probe for the 5′-most region of the first, 7.5 kb-long *Ubx* intron, which should detect the first ∼10 min of *Ubx* transcription (Figure 3, D–G). In some nuclei we detected *Ubx* and *Cbx hybrid* transcripts that appeared to derive from the same DNA molecule (Figure 3, F and G), thus arguing against transcriptional interference by *Cbx hybrid* transcript. One caveat to this statement is that it is possible that some of the imaged chromosomes contained replicated sister chromatids and so the overlap of *Cbx hybrid* and *Ubx* transcript signals could be due to adjacent DNA molecules in cell cycle G2.

### CRISPR-mediated knockout of *lncRNA:PS4* has no effect on Ubx expression, but it interacts genetically with the *Ubx^Cbx-1^* mutation in developing wings

**Figure 4:**
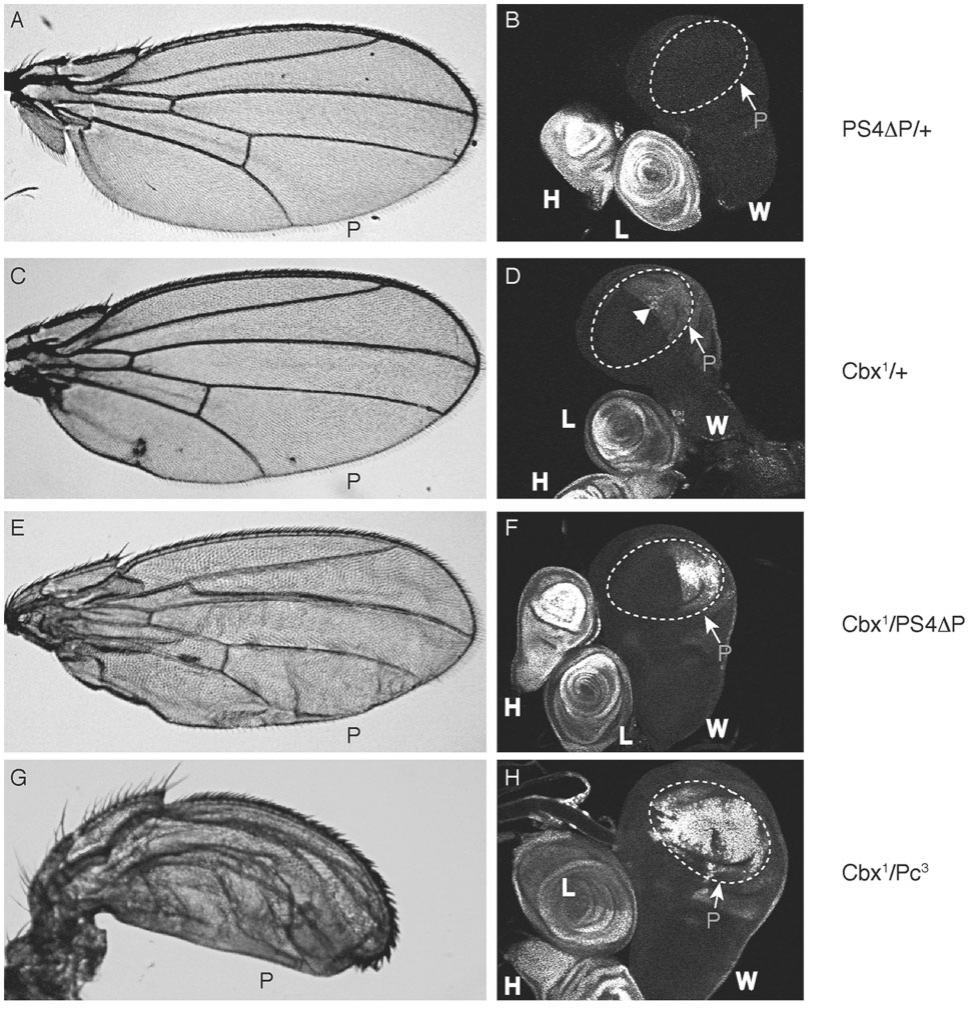
*lncRNA:PS4* genetically interacts with the *Ubx^Cbx-1^* mutation in wing discs and adult wings. (A, C, E, G) adult wings. P indicates the posterior margin of the wing. (B, D, F, H) wing (W), haltere (H), and leg (L) discs, immunostained for Ubx protein. The areas of the wing discs that will contribute to the adult wings are outlined by a dotted line; the posterior compartment of the prospective wing is labeled as P. (A, B) Flies heterozygous for the deletion of *lncRNA:PS4* promoters (*PS4ΔP*/*TM3-Sb* in A and *PS4ΔP*/*TM3Sb, Kr-GFP* in B). Note the normal wing phenotype (A) and lack of activation of Ubx protein expression in the wing primordia, compared with strong Ubx expression in the haltere and leg discs. (C, D) *Ubx^Cbx-1^* heterozygous flies (*Ubx^Cbx-1^/TM3-Sb* in C and *TM3Sb, Kr-GFP* in D). The posterior wing is slightly deformed, and slight activation of Ubx protein expression is detected in the posterior wing disc (arrowhead). (E, F) Flies heterozygous for *Ubx^Cbx-1^* and the *lncRNA:PS4* promoter deletion*.* Note the stronger abnormalities in the posterior part of the adult wing compared with *Ubx^Cbx-1^* and a stronger activation of Ubx protein expression in the posterior wing disc. (G, H) For comparison, the adult wings and stained wing discs in *Ubx^Cbx-1^*/*Polycomb* (Pc^3^) heterozygotes. Note the transformation of the wing toward haltere and the strong activation of Ubx protein throughout anterior and posterior compartments of the wing disc.

To further test the function of the *lncRNA:PS4* transcript, we generated a CRISPR/Cas9-mediated deletion of 324 bp that removed both of its promoters. Whole-mount *in situ* hybridization demonstrated that *lncRNA:PS4* transcription was largely eliminated in the homozygous mutants (Supplementary Figure S5). Next, we investigated the effects of the *lncRNA:PS4* mutation on *Ubx* expression. In embryos homozygous for the promoter deletion, the abundance and pattern of *Ubx* transcripts were the same as in wild-type embryos. Only 5–6% of the *lncRNA:PS4* homozygous mutant flies emerged as adults (with most homozygotes dying in late larva and pupa stage), apparently because of an additional mutation elsewhere on the third chromosome, since *trans*-heterozygotes of the *lncRNA:PS4* promoter deletion over a deletion of the *Ubx* locus [*Df(3R)Ubx^109^*], resulted in viable adults at approximately wild-type frequencies. We then examined if the deletion of *lncRNA:PS4* promoters resulted in ectopic activation of Ubx protein in larval wing discs, or in wing abnormalities. The wing discs and the wings of the surviving homozygous adults were similar to those of flies heterozygous for the deletion and contained no wing deformations (Figure 4A), or ectopic activation of Ubx protein in wing discs (Figure 4B).

We also examined whether the *lncRNA:PS4* promoter deletion mutation interacted genetically with the *Ubx^Cbx-1^* mutation. In wing discs heterozygous for *Ubx^Cbx-1^*, we observed ectopic Ubx protein in the posterior region of the disc (Figure 4D), as previously described (White and Akam 1985). In our hands, adults that were heterozygous for the *Ubx^Cbx-1^* insertion had only slight defects in wing morphology (Figure 4C), likely because of accumulation of modifiers in this strain. As reported previously (Castelli-Gair *et al.* 1992), *Polycomb* (*Pc*^3^) mutants enhance the amount and extent of *Ubx* expression in the wing disc of *Ubx^Cbx-1^* mutants (Figure 4H), and have a stronger transformation of wings toward halteres (Figure 4G). The *lncRNA:PS4* promoter deletion also enhances the adult wing phenotype of *Ubx^Cbx-1^* (Figure 4E), albeit much more mildly than *Pc*^3^. The ectopic expression of Ubx protein in the posterior compartment of wing discs from *Ubx^Cbx-1^*/*lncRNA:PS4* larvae was also enhanced (Figure 4F), compared with that seen in discs of larvae heterozygous for *Ubx^Cbx-1^*.

## Discussion

Although in embryos the antisense *lncRNA:PS4* transcripts are expressed in cells that are largely nonoverlapping with *Ubx*, the function of *lncRNA:PS4* seems to have little if any effect on the pattern of *Ubx* expression in otherwise wild-type animals. Two lines of evidence support this conclusion. First, a small deletion that eliminates almost all *lncRNA:PS4* transcription has no detectable effect on *Ubx* expression in embryos and in imaginal discs. Second, the abundant transcription of 3′ *lncRNA:PS4* sequences driven by the *Ubx^Cbx-1^* insertion in posterior regions of the embryo has no detectable repressive effect on *Ubx* transcription in such posterior regions.

In animals that are heterozygous for *Ubx^Cbx-1^* and *lncRNA:PS4* promoter mutations there is an enhancement of the *Ubx^Cbx-1^* phenotype, and one can interpret this as noncomplementation between these two alleles, suggesting that *lncRNA:PS4* has a very subtle repressive effect on *Ubx* transcription in wing discs which can only be observed in a *Ubx^Cbx-1^* mutant background. One possible mechanism by which the *lncRNA:PS4* region may provide this subtle repression is by helping to set a repressive chromatin state in the cells in which it is expressed, as was suggested for the *bxd* lncRNA (Pease *et al.* 2013).

One puzzle is how the enhancer/promoter for *lncRNA:PS4* that resides in the *Ubx* locus is able to activate transcription within parasegment 4, whereas the rest of the *Ubx* locus is repressed in parasegment 4. This seems potentially inconsistent with the “open for business” model, in which the *Ubx* locus as a whole has been observed to be associated with H3K27 trimethylation repressive modifications in the bulk of embryonic parasegment 4 cells (Bowman *et al.* 2014; Maeda and Karch 2015). Those modifications recruit Polycomb group proteins that maintain the off state of *Ubx* in those cells. However the expression of *lncRNA:PS4* is transient in most cells of parasegment 4, and may help recruit Polycomb group complexes at very early embryonic stages that then quantitatively assist in the maintenance of *Ubx* repression in the cells of that parasegment through later stages of embryogenesis and beyond.

The *lncRNA:PS4* promoters are both contained within a predicted Polycomb Repression Element (Supplementary Figure S1; Négre *et al.* 2011). It may be that either *lncRNA:PS4* transcripts themselves contribute to the function of this PRE or that they are merely a signal associated with the PRE regulatory element, as noncoding RNAs are often produced in the vicinity of *cis*-regulatory elements (Natoli and Andrau 2012). If this is the case, it is possible that the *Ubx^Cbx-1^*-dependent effect of *lncRNA:PS4* promoter deletions on *Ubx* expression in the wing is due to the sequences removed from the predicted PRE, and not due to elimination of *lncRNA:*PS4 transcripts *per se*. It is also possible that *lncRNA:PS4* transcripts have a repressive effect on *Ubx* in some cells that we did not test, but an important effect of *lncRNA:PS4* on early embryonic patterning via regulation of *Ubx* is not supported by our results.

In some respects, the *Hox*-encoded *lncRNA:PS4* transcripts resemble that of the *Hox* cluster-encoded *HOTAIR* transcripts of mice. Different studies have proposed that *HOTAIR* either has an important (Rinn *et al.* 2007; Li *et al.* 2013), or largely unimportant (Amândio *et al.* 2016; Schorderet and Duboule 2011) role in regulating *Hox* gene expression and embryonic patterning. As proposed by Amândio *et al.* (2016), these inconsistent findings might be explained by the different genetic backgrounds in which *HOTAIR* mutant alleles were tested. As described here, the function of *lncRNA:PS4* transcripts also seem sensitive to genetic background, as *lncRNA:PS4* mutant animals only exhibit ectopic expression of *Ubx* and abnormal wing shapes in the background of a *Ubx^Cbx-1^* mutant allele. 

## Data availability

Strains and plasmids are available upon request. Reagents and primer sequences not included in the *Materials and Methods* section are listed in the Reagents Table. The GenBank accession number of *lncRNA:PS4* is OK501974.

Supplementary material is available at *G3* online.

## Funding

This research was supported by NIH grant R01HD28315 to W.M. 

## Conflicts of interest

The authors declare that there is no conflict of interest.

## Supplementary Material

jkab374_Supplementary_Figures-Tables

jkab374_Supplementary_Data
