# Supplementary material for: The expression of Drosophila melanogaster Hox gene Ultrabithorax is not overtly regulated by the intronic long noncoding RNA lncRNA:PS4 in a wild-type genetic background
Source: G3 (Bethesda). 2021 Nov 15;12(1):jkab374. doi: 10.1093/g3journal/jkab374 (PMC8727962; doi:10.1093/g3journal/jkab374)
Supplement: jkab374_Supplementary_Figures-Tables [file jkab374_supplementary_figures-tables.pdf]

Supplemental materials

Supplemental figure legends

Supplemental figure 1. 5' end of *lncRNA:PS4* transcriptional unit, as detected by 5' RACE. Sequences from the second intron of *Ubx,* with the two putative transcriptional starts of *lncRNA:PS4* highlighted in purple (the first (5'-most) nucleotide detected by 5' RACE). P1 and P2 indicate the upstream and the downstream promoters, respectively. The following putative regulatory elements were also noted in the adjacent sequences: a solid underline indicates a sequence comprising a match to the *Drosophila* InR promoter consensus sequence, and the dotted underline indicates a weaker match to an InR site (Ngoc et al., 2019). Green is a putative DPE promoter sequence, located 29 (and not the canonical 28) nucleotides downstream of the second transcriptional start site (Ngoc et al., 2019). Red text indicates the approximate 5' and 3' boundaries of a predicted Polycomb repression element (PRE), characterized by ChIP-detected binding of Rpd3 (HDAC1) or HDAC4 along with overlapping H3K27me3 (but not H3K4me3) histone modifications (Négre et al., 2011).

Supplemental figure 2. Transcription of *lncRNA:PS4* and *Ubx* occurs in adjacent, largely non overlapping domains. Whole mount *in situ* hybridization of embryos (stages 5-10), detecting *lncRNA:PS4* (*X* probe, green) and *Ubx* (cDNA probe, magenta).

Supplemental Figure 3. High-resolution images of *lncRNA:PS4* transcription and its spatial relationship with *Ubx* and *wingless* expression domains. A-C: *in situ* hybridization of an early stage 6 wild type embryo probed with *wingless* (blue), *Ubx* cDNA probe (red) and *lncRNA:PS4* (green, detected with probe *X*) antisense probes (imaged at 40X magnification). B and D are enlargements of the areas marked with the white square in A and C,respectively. Parasegments (PS) 4, 5, and 6 are indicated and marked with brackets.

Supplemental figure 4. Transcription of *Cbx hybrid* transcript extends into the 5' end of the first intron of *Ubx*, but the expression pattern of *Ubx* does not change. A, B. *in situ* hybridization with the probe against the strand expressing *lncRNA:PS4* RNA, corresponding to the first 1.5 kb of the first intron of *Ubx* gene (magenta) and with *wingless* probe (blue). A. In wild type embryos, *lncRNA:PS4* transcription does not extend to the beginning of the first intron of *Ubx*. B. In Ubx^{Cbx-1} mutant embryos, strong transcription of the *Cbx hybrid* transcript can be detected with a probe corresponding to the beginning of the first intron of *Ubx*. C, D. Stage 6 embryos (ventral view) hybridized with *Ubx* cDNA, *lncRNA:PS4* (using probe *X*), and *wingless* cDNA probes. (C) In wild type embryos, the anterior boundary of *Ubx* transcription is in parasegment 6. (D) In Ubx^{Cbx-1} mutant embryo, the anterior boundary of *Ubx* is preserved and the posterior expression is not suppressed despite the expression of the *Cbx hybrid* transcript in posterior regions.

Supplemental figure 5. Targeted deletion of the two promoters of *lncRNA:PS4* results in depletion of its transcripts. Stage 7 wild type embryo (A) and stage 7 embryo homozygous for the *lncRNA:PS4* promoters' deletion (B). (C) and (D) are enlargements of the regions indicated with blue rectangles in (A) and (B). *lncRNA:PS4* signal was detected using tyramide amplification.

Supplementary Table 1. Quantitative RT-PCR for imaginal disc expression of *lncRNA:PS4*. Two different gene-specific primer pairs were used to detect the abundance of transcripts (Ct values) for *lncRNA:PS4* and a positive control *Ultrabithorax* (*Ubx*) in wing or haltere/leg imaginal discs taken from third instar larvae just prior to pupariation. Fewer Ct (Cycle threshold) values denote higher levels of expression. Negative (no reverse transcriptase) controls were somewhat variable, but averaged 38 Ct. Note that although *Ubx* transcripts are not expressed in the primoridia of the part of the wing imaginal disc that gives rise to the actual wing, they are expressed in the peripodial membrane cells of the wing imaginal disc, hence the Ct values that indicate *Ubx* transcripts in the wing imaginal discs.

Supplemental Figure 1

Supplemental figure 1

TTATAACTTCGGATATCTTACAAAAGCTAACATCTATAAGATATAACACCCATACAGGTAAATCCAACCAAGTCTCT

TATCTGAGATTTCCTAACGACTCCAGATAATCTTAACTGGTATAACAATCTTCTTTGCCTCACTAACCTTGGTCTAA

TCAGAAGAATTATCTCACCTCTGCGGACTCCCATTGACAGCTGCGAATATCGTAAACGGTCTACGATTACCTGTA

TTGTTCCAATTCGAATAAAGGTGAGATTGAGATTCATTTCGCCGAGAGCAGATAAATTTACTCCCAAAGCAACCA

CTAAACCGGCTGAGCGAAATGATGATCGATCACCAGGTGAGCGAAAGAGAGGAGCCAGATAAATAGAGAGAAC

GCGCACAGAGAGCAGATGAATAAGAAGCCGAGAGAAAGAACGATCGCATACCTGTGCAGTGAGCAAGCAACTT

P1

ATTCTGTGAATCAGGTGAAGATCACTGAAGATTTCCAGCCATCTGACATTTCCAAAATGGGTTCACGCACGCAC

P2

ACCAGCACACTTGTTCCTATGGGCGATCAGTCACTTGTTTCTATGAGCGATCACCACCAGGTTCATTTCATTTGC

CCAACGGTCGCGGACATTGTCATGACGTTTGCTCTTTTTTCGGGTCTGCGCGATCAGCTGATCATGATGGCCG

CCAAAAGCAGGTTTTCAGCTCAACCGAGCATTATTAATCATAAGCCGAAAGAGAGGGCACCTCCAACAGAGTGA

GTGGGAAAGACAACAAACGCTTTTCAAATACCACAAAATTTATGGCTTGAAGCCAAGCCGACGGCAAGCA

Figure 2.Overlay of *lncRNA:PS4* expression pattern (green) and with *Ubx transcripts* (magenta)

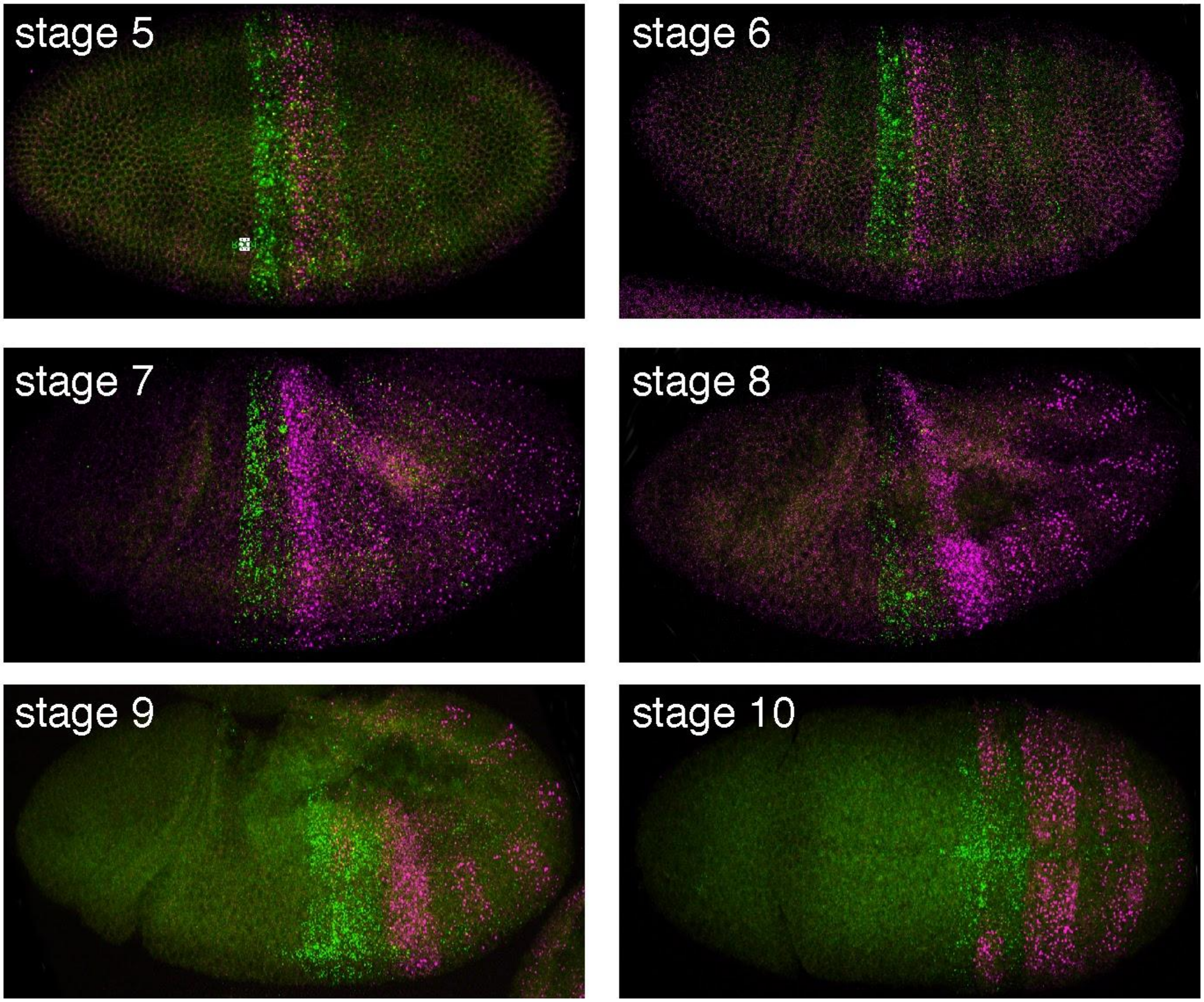

Supplemental Figure 3

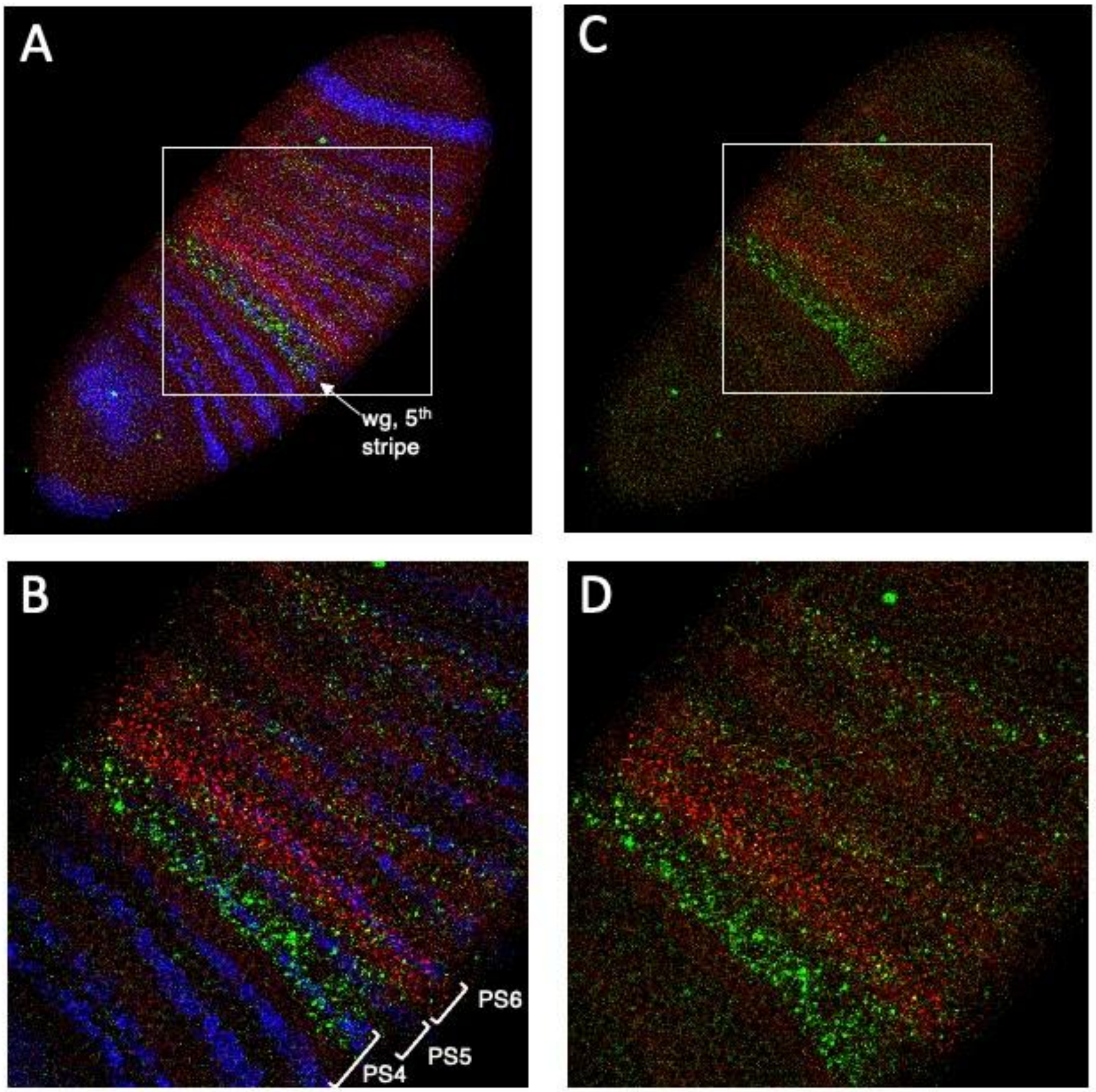

Supplemental Figure 4

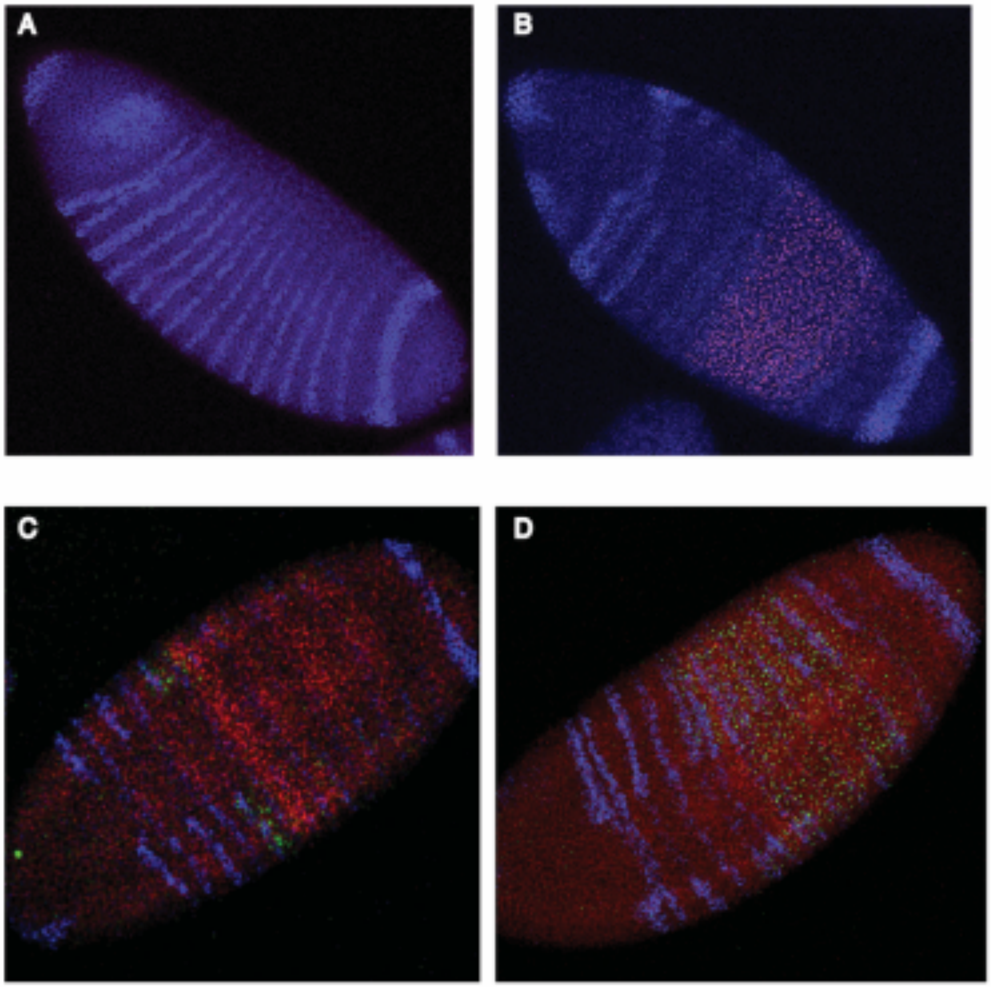

Supplemental Figure 5

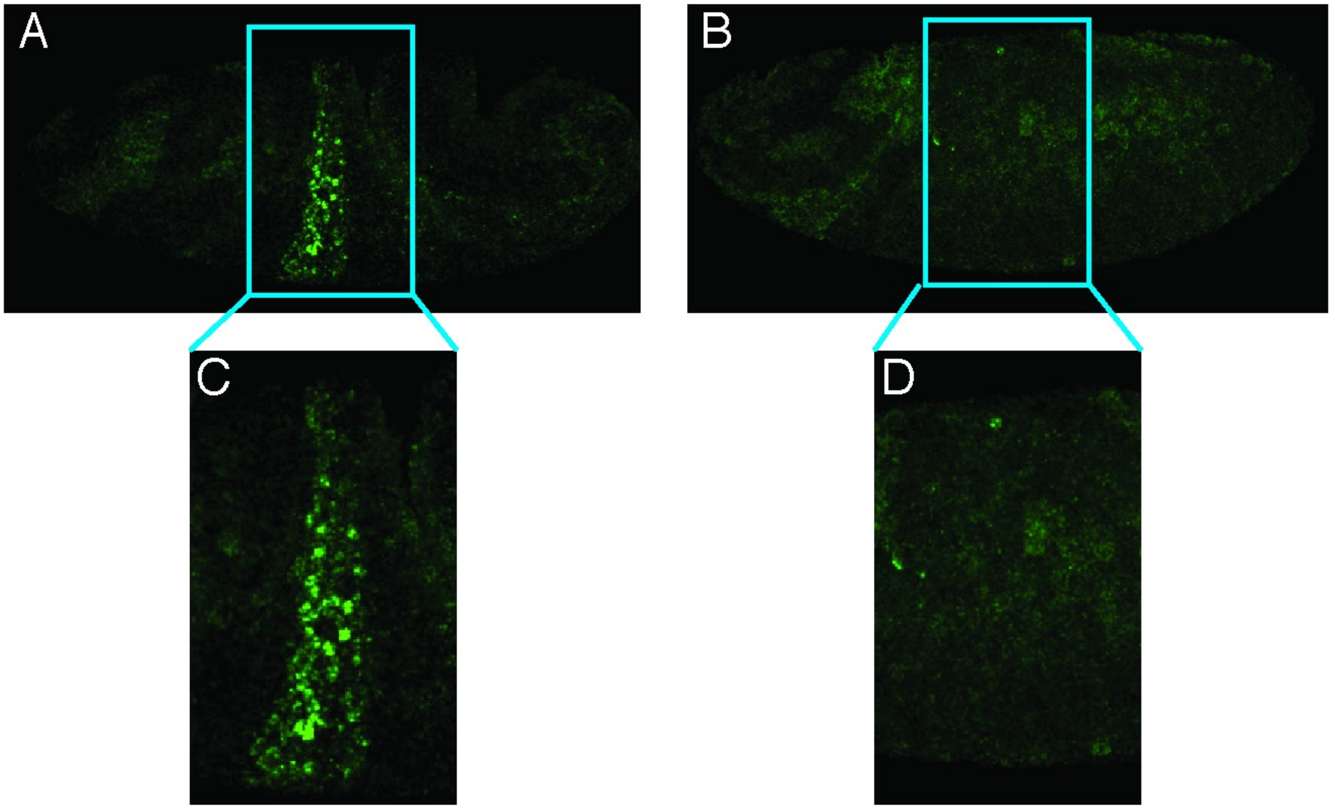

Supplementary Table 1.

	**Wing discs Ct values**	**Haltere and leg discs Ct values**
lncRNA:PS4		
Primer pair 1	32 cycles	28 cycles
Primer pair 2	32 cycles	28 cycles
***Ultrabithorax* control**		
Primer pair 1	27 cycles	27 cycles
Primer pair 2	28 cycles	26 cycles
